# Regulation of hypoxia inducible factor-1α expression by the alteration of redox status in HepG2 cells

**DOI:** 10.1186/1756-9966-30-61

**Published:** 2011-05-19

**Authors:** Wen-sen Jin, Zhao-lu Kong, Zhi-fen Shen, Yi-zun Jin, Wu-kui Zhang, Guang-fu Chen

**Affiliations:** 1 Teaching & Research Section of Nuclear Medicine, An-hui Medical University, Hefei, China; 2 Eighth Laboratory, Institute of Radiation Medicine, Fudan University, Shanghai, China

**Keywords:** Hypoxia, Redox, Multidrug resistance, HepG2

## Abstract

Hypoxia inducible factor-1 (HIF-1) has been considered as a critical transcriptional factor in response to hypoxia. It can increase P-glycoprotein (P-Gp) thus generating the resistant effect to chemotherapy. At present, the mechanism regulating HIF-1α is still not fully clear in hypoxic tumor cells. Intracellular redox status is closely correlated with hypoxic micro-environment, so we investigate whether alterations in the cellular redox status lead to the changes of HIF-1α expression. HepG2 cells were exposed to Buthionine sulphoximine (BSO) for 12 h prior to hypoxia treatment. The level of HIF-1α expression was measured by Western blot and immunocytochemistry assays. Reduce glutathione (GSH) concentrations in hypoxic cells were determined using glutathione reductase/5,5^'^-dithiobis-(2-nitrob-enzoic acid) (DTNB) recycling assay. To further confirm the effect of intracellular redox status on HIF-1α expression, *N-*acetylcysteine (NAC) was added to culture cells for 8 h before the hypoxia treatment. The levels of multidrug resistance gene-1 (MDR-1) and erythropoietin (EPO) mRNA targeted by HIF-1α in hypoxic cells were further determined with RT-PCR, and then the expression of P-Gp protein was observed by Western blotting. The results showed that BSO pretreatment down-regulated HIF-1α and the effect was concentration-dependent, on the other hand, the increases of intracellular GSH contents by NAC could partly elevate the levels of HIF-1α expression. The levels of P-Gp (MDR-1) and EPO were concomitant with the trend of HIF-1α expression. Therefore, our data indicate that the changes of redox status in hypoxic cells may regulate HIF-1α expression and provide valuable information on tumor chemotherapy.

## Introduction

The majority of transcriptional responses in cells to hypoxia are mediated by hypoxia inducible factor-1(HIF-1), a heterodimeric protein that consists of the steadily expressed HIF-1β/ARNT and the highly regulated HIF-1α subunits. The HIF-1α subunit, under normoxic conditions, is hydroxylated by prolyl hydroxylasamses (PHDs) at praline residues 402 and 564 in the oxygen-dependent degradation (ODD). Then it is targeted for proteasome-mediated degradation through a protein ubiquitin ligase complex containing the product of the von Hippel Lindau tumor suppressor (pVHL) [1,2]. Many data revealed that there was a rapid biodegradation of HIF-1α protein within 5-10 min when hypoxic condition was changed into normoxic condition; furthermore the expression of HIF-1α protein was undetectable by the end of 30 min in normoxia [3,4]. In contrast, the degradation pathway is blocked when cells are exposed to a hypoxic environment, thereby allowing HIF-1α to accumulate and migrate to the nucleus, where more than 100 genes have been identified as direct targets of HIF-1α [5,6]. Among these genes, many are responsible for the physiological or pathophysiological activities of hypoxic cells, including cell survival, glucose metabolism, glycolysis and therapeutic resistance [7-9].

The expression level of HIF-1α is regulated by different factors involving cell signal transduction pathway, cytokines, heat-shock protein 90, reaction oxygen (ROS) and nitric oxide (NO) [10-13]. It is well known that intracellular antioxidant systems, such as reduce glutathione (GSH), superoxide dismutase, glutathione peroxide, etc, can scavenge the excess ROS and sustain the redox equilibrium in cells [14]. Studies have shown that GSH play a role in protecting cells from oxide free radicals, ROS and nitrogen radicals [15-17]. It is, therefore, possible that the level of HIF-1α expression may be regulated by modifying the redox status of hypoxic cells.

To test this hypothesis, we used redox reagents to alter the contents of intracellular GSH, which resulted in the changes of redox status in hypoxic cells, then to evaluate whether the modifications of redox status in hypoxic cells can regulate HIF-1α protein levels.

## Materials and methods

### Cell viability assay (MTT)

The effect of BSO on tumor cell growth was determined using an MTT colorimetric assay [18]. Cells were seeded in 96-well plates at a density of 5 × 10^3 ^cells per well. They were, then, treated with different concentrations of BSO for 12 h. Furthermore, the medium was replaced with fresh medium allowing cells to be continuously grown up to 72 h. The 3-(4,5-dimethylthiazol-2-yl)-2,5-diphenyltetrazo-lium bromide (MTT, Sigma) dye was added to a final concentration of 50 mg/ml and cells were subsequently incubated for another 4 h at 37°C. The media containing residual MTT dye was carefully aspirated from each of the wells and 200 μl DMSO was added to each well to dissolve the reduced formazan dye. The effect of BSO on the growth of cells was determined from differences in absorbance. The fraction of cells viability was calculated by comparing the optical absorbance of culture given a BSO treatment with that of the untreated control.

### Cells culture and treatment

HepG2 cells (Cell Bank, Chinese Academy of Sciences) were cultured in RPMI-1640 medium (GIBCO BAL, USA) supplemented with 10% FBS, penicillin (100 U/ml), streptomycin (100 μg/ml) at 37°C in an incubator containing humid atmosphere of 95% air and 5%CO_2 _and propagated according to protocol given by the American Type Culture Collection. Hypoxic treatment was in a controlled chamber maintained with 1% O_2_, 99%N_2 _for 4 h. The medium was changed prior to experiments. To investigate the effect of redox state on the hypoxia induction of HIF-1α expression, the cells were cultivated for 12 h in the absence or presence of 50 μM, 100 μM and 200 μM DL-Buthionine sulphoximine (BSO, Sigma, USA) before the 4-h hypoxia treatment. In addition, 5 mM *N-*acetylcysteine (NAC) (Sigma, USA), an antioxidant and GSH precursor, was used to culture cells for 8 h before hypoxia to further confirm the mechanism of BSO modulating the expression of HIF-1α by the changes of micro-environment redox status in the cells.

### Intracellular GSH assay

After the triplicate samples of 10^6 ^cells were treated under different conditions, The GSH/GSSG ratios were measured with the glutathione reductase/5,5^'^-dithiobis -(2-nitrobenzoic acid) (DTNB) recycling assay kit (Beyotime, China) under the methods recommended by the manufacturer. The standard sample and checking sample cuvettes were placed into a dual-beam spectrophotometer, and the increases in absorbance at 412 nm were followed as a function of time. The standard curves of total glutathione and GSSG concentrations were fitted with absorbance, followed by determining the concentration of checking samples. Concentrations were converted to nmol/mg protein, and reduced GSH concentrations were obtained by subtracting two times GSSG from total glutathione. Finally, GSH/GSSG ratio, with different treatment, was calculated through cellular GSH concentration divided by GSSG concentration.

### RNA purification

Cells were lysed by TRIzol Reagent and RNA was extracted according to manufacturer's instruction (Sangon, China). To avoid genomic DNA contamination, extracted RNA was then purified with the RNeasy kit (Invitrogen, USA). The quantity and quality of RNA was determined by the OD measurement at 260 and 280 nm. The integrity of RNA was checked by visual inspection of the two rRNAs 28S and 18S on an agarose gel.

### RT-PCR

Two micrograms RNA was used for cDNA synthesis using Olig-(dt)_18 _as primer and AMV reverse transcriptase. The RT reaction was started with 10 min incubation at room temperature, and then at 42°C for 60 min, followed by 10 min at 70°C to terminate the reaction. Subsequently, a 2 μl aliquot of cDNA was amplified by PCR in a total volume of 25 μl containing 2.5 μl 10 × PCR buffer (0.2 M Tris-HCl, pH 8.4, 0.5 M KCl), 0.2 mM dNTP mix, 1.5 mM MgCl_2_, 0.2 μM of each primer and 1.25 units of Platinum Taq DNA polymerase (Invitrogen, USA). The thermal cycler was set to run at 95°C for 5 min, 30 cycles of 94°C for 30 s, 52°C for 30 s, 72°C for 1 min, and a final extension of 72°C for 10 min. The primers specific for multidrug resistance gene-1 (MDR-1) and erythropoietin (EPO) (MDR-1 upstream: 5'-CCA ATGATGCTGCTCAAGTT-3'; downstream: 5'-GTTCAAACTTCTGCTCCT GA-3'; 297-bp fragment; EPO upstream: 5'-ATATCACTGTCCCAGACACC-3'; downstream: 5'-AGTGATTGTTCGGAGTGGAG-3'; 290-bp fragment) were used, and for β-actin (upstream: 5'-GTTGCGTTACACCCTTTCTTG-3'; downstream: 5'-GACTGCTGT CACCTTCACCGT-3'; 157-bp fragment) were as control. PCR products were analyzed by electrophoresis in 1.2% agarose gel. The specific bands were visualized with ethidium bromide and digitally photographed under ultraviolet light, furthermore scanned using Gel Documentation System 920 (Nucleo Tech, San Mateo, CA). Gene expression was calculated as the ratio of mean band density of analyzed specific products to that of the internal standard (β-actin).

### Western blot analysis of HIF-1α expression

Cells were scraped off from culture flasks and lysed in lysis buffer containing 10% glycerol, 10mMTris-HCL(PH 6.8), 1%SDS, 5 mM dithiothreitol (DTT) and 1× complete protease inhibitor cocktail (Sigma, USA). The method of Bradford was used to assay concentrations of protein in diverse samples. Protein concentration was measured using an auto multifunction microplate reader. Fifty micrograms of cellular proteins were separated by 8% polyacrylamide-SDS inconsecutive gel electrophoresis. The separated proteins were electrophoretically transferred to polyvinylidene difluoride membrane. Membranes were blocked with a 5% skim milk in Tris-buffered saline (TBS) containing 0.1% Tween 20 at room temperature for 1 h and then incubated with mouse anti-human monoclonal HIF-1α (Abcam, USA) at a 1:500 dilusion and P-glycoprotein (P-Gp) antibody (Abcam, USA) at a 1:200 dilusion overnight at 4°C, followed by goat anti-mouse IgG for 1 h at room temperature. Signals were detected with enhanced chemiluminescence (ECL plus, Amersham, USA). Microtubule protein (Tubulin, Abcam, USA) at a 1:1000 dilution was used as internal control to observe the changes of HIF-1α and MDR-1 bands.

### Immunocytochemistry analysis of HIF-1α expression

Cells grew on coverslips in 6-well culture dishes to approach 70% confluence; they were then treated with BSO and NAC as above description, following 4 h hypoxic treatment. After the medium was completely removed by suction, the cells were rinsed briefly with phosphate buffer saline (PBS). Then, 4% Formaldehyde was used to fix the cells on coverslips for 10 min at room temperature, and then methanol fixed the cells for 10 min at -20°C. To utilize 0.5% TritonX-100 enhanced permeabilizations of the cells for 10 min at room temperature. The coverclips were pre-incubated with 3% hydrogen peroxide (H_2_O_2_)-methyl alcohol mix solution for 10 min to block endogenous peroxidase activity, followed by incubation for 30 min with block solution at room temperature. Cells were incubated with primary antibody, a mouse anti-human monoclonal HIF-1α antibody, at a 1:1300 dilution overnight at 4°C. Then cells were incubated with biotinylated secondary antibody, followed by a routine immunoperoxidase processing. After washed twice with PBS, these coverslips were developed using diaminobenzidine (DAB) as a chromogen, rinsed, gradient dehydrated by alcohol, and then mounted on slides. The coverslips without primary antibody treatment was regarded as the negative control. H-score values were used as a semi-quantitative evaluation for immunocytochemistry [19].

### Statistical analysis

Data were reported as the means ± SEM of three separate experiments. Statistical significance was measured by independent sample *t *test and analysis of variance. A value of *p *< 0.05 was considered as statistically significant.

## Results

### Selection of sublethal concentration of BSO

**Figure 1:**
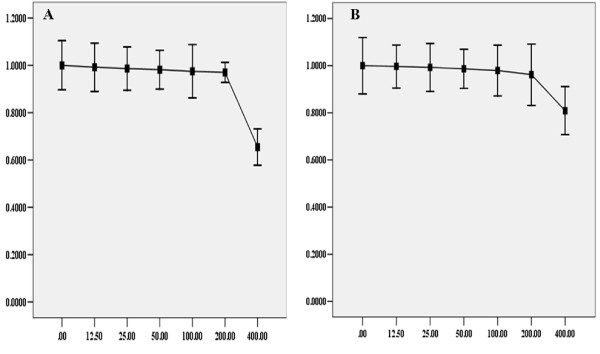
**Toxicity of BSO on HepG2 cells**. Under normoxic or hypoxic condition, HepG2 cells were treated with different concentration of BSO for 12 h before subjected to the MTT assay. The viability was calculated by subtracting the background absorbance and divided by the control absorbance. Both normoxia and hypoxia, the results showed that there was not significance in the decrease of cells viability until the concentration of BSO was at 400 μM. The change of cells viability, under normoxia or hypoxia, was displayed in Diagram A and Diagram B respectively.

In order to select the appropriate concentration of BSO for the study, a 12 h dose-response study was conducted by exposing cells to different concentrations of BSO. Cell viability was measured by the MTT assay. The results showed that there was not significant decrease in viability over a 12 h exposure to BSO concentration ranging from 12.5 to 200 μM (Figure 1). In subsequent studies, the concentrations of BSO used were set at 50, 100, 200 μM.

### Variations of intracellular redox status

**Figure 2:**
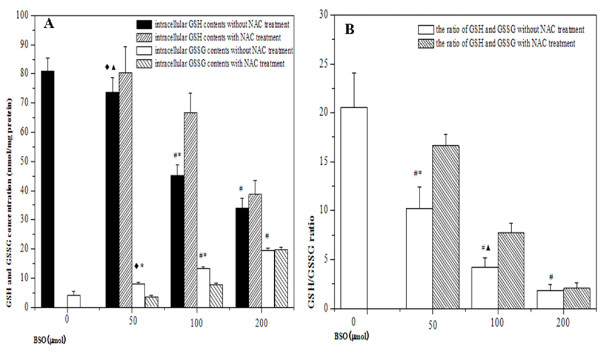
**The changes of redox status in hypoxic cells by different pretreatment**. (A) showed the alteration of intracellular GSH and GSSG contents in HepG2 cells under hypoxic condition; (B) showed the ratios of GSH and GSSG in HepG2 cells under hypoxic condition. (^◆^*p *< 0.05, ^#^*p *< 0.01, as compared with hypoxia control; ^▲^*p *< 0.05, *p < 0.01, as compared with the cells by NAC treatment).

As shown in Figure 2, BSO treatment led to significant reduction of intracellular GSH level and the effect was in a concentration-dependent manner. Intracellular GSSG contents were increased concomitant with BSO concentrations, resulting to subsequent reductions of GSH/GSSG ratios. The declines of GSH level were partially restored from hypoxic cells by the addition of 5 mM NAC prior to hypoxia. Compared with the cells in the absence of NAC, there was an increase in GSH/GSSG ratio in the presence of 5 mM NAC. It indicated that BSO inhibited the accumulation of GSH in cells, but the effect could be partially reversed by NAC treatment.

### Effect redox status on HIF-1α expression

**Figure 3:**
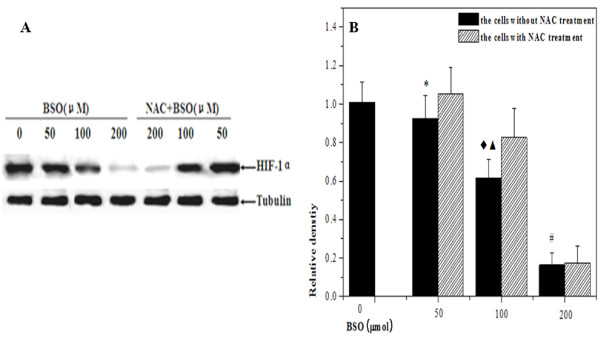
**The change of HIF-1α proteins in HepG2 cells under hypoxic condition by Western blotting measurement**. (A) The representative gel picture was taken from three separate experiments. (B) Compared with hypoxic control, the expression of HIF-1α was reduced in BSO concentration-dependent manner, and the analysis of relative densities showed that there was statistical difference the experimental cells by 100 and 200 μM BSO pretreatment respectively (^◆^*p *< 0.05, ^#^*p *< 0.01). After NAC incubation, the expression of HIF-1α was elevated again, and there were significant difference between the group with 100 μM NAC treatment and that without NAC treatment (^▲^*P *< 0.01).

HIF-1α protein levels were measured using Western blot after BSO pretreatment. When BSO concentration reached at 50 μM, the down-regulation of HIF-1α expression, under the hypoxia condition, was observed in HepG2 cells. It is then very clear that HIF-1α proteins in hypoxic cells were significantly decreased with BSO concentrations gradually increasing. In addition, the inhibition of HIF-1α expression was reversed by 5 mM NAC supplement. However, we also found that NAC failed to elevate the level of HIF-1α expression inhibited by BSO concentration at 200 μM. These results were shown in Figure 3

**Figure 4:**
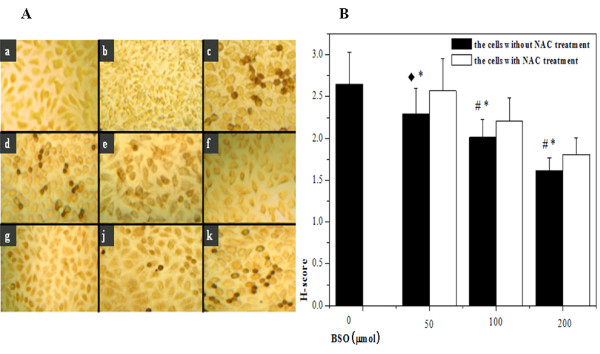
**The change of HIF-1α expression by ICC assay**. (A) The picture of ICC was shown. a: negative control; b: normoxic control; c: hypoxic control; d: the hypoxic cells by 50 μM BSO pretreatment; e: the hypoxic cells by 100 μM BSO pretreatment; f: the hypoxic cells by 200 μM BSO pretreatment; g: the hypoxic cells by 50 μM BSO + 5 mM NAC pretreatment; j: the hypoxic cells by 100 μM BSO + 5 mM NAC pretreatment; k: the hypoxic cells by 200 μM BSO + 5 mM NAC pretreatment. (B) The results of statistical analysis were shown with H-score values of semi-quantitative evaluations. (^◆^*P *<0.05, ^#^*p *< 0.01, compared with hypoxic control; *P <0.05, compared with the hypoxic cells by 5 mM NAC pretreatment).

To further verify the effect of redox status on HIF-1α levels, we detected the expressions of HIF-1α proteins by using immunocytochemistry technique (ICC). As shown in Figure 4, cells showed more negative staining than control group after BSO pretreatment and NAC decreased the inhibition. The results were basically consistent with Western blot result.

### Changes of genes targeted by HIF-1

**Figure 5:**
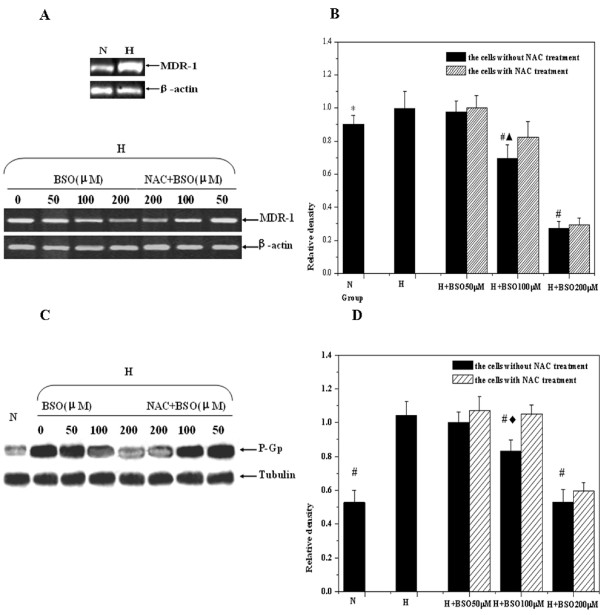
**The changes of MDR-1 expressions by RT-PCR and Western blotting measurement**. Letter N means the cells under normoxic condition; Letter H means the cells under hypoxic condition: (A) The representative gel picture was taken from three separate RT-PCR experiments. (B) Compared with hypoxic control, the analysis of relative densities showed that there was statistical difference the experimental cells by 100 and 200 μM BSO pretreatment respectively (^#^*p *< 0.01). After NAC incubation, the expression of MDR-1 was elevated again, and there were significant difference between the group with 100 μM NAC treatment and that without NAC treatment (^▲^*P *< 0.05). (C) The representative gel picture was taken from three separate Western blotting experiments. (D) Compared with hypoxic control, the analysis of relative densities showed that there was statistical difference the experimental cells by 100 and 200 μM BSO pretreatment respectively (^#^*p *< 0.01). After NAC incubation, the expression of MDR-1 was elevated again, and there were significant difference between the group with 100 μM NAC treatment and that without NAC treatment (^◆^*P *< 0.01).

**Figure 6:**
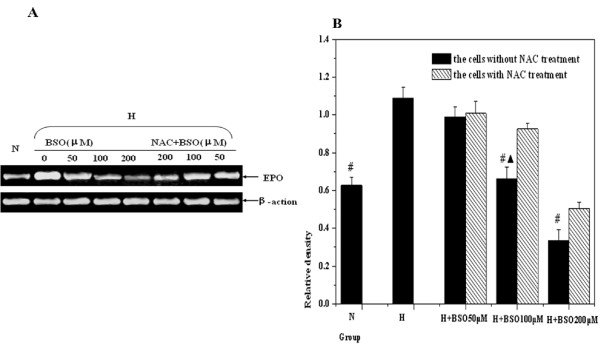
**The changes of EPO expressions by RT-PCR measurement**. Letter N means the cells under normoxic condition; Letter H means the cells under hypoxic condition: (A) The representative gel picture was taken from three separate RT-PCR experiments. (B) Compared with hypoxic control, the analysis of relative densities showed that there was statistical difference the experimental cells by 100 and 200 μM BSO pretreatment respectively (^#^*p *< 0.01). After NAC incubation, the expression of EPO was elevated again, and there were significant difference between the group with 100 μM NAC treatment and that without NAC treatment (^▲^*P *< 0.01).

The levels of MDR-1 and EPO transcription were detected through semi-quantitative RT-PCR. The results displayed that the levels of MDR-1 and EPO mRNA were declined in hypoxic cells when BSO concentration was at 50 μM, but it wasn't shown that there was a statistical significance at the MDR-1 and EPO mRNA of 50 μM BSO pretreatment compared with those of the hypoxic control. Concomitant with the increases of BSO concentrations, the levels of MDR-1 and EPO mRNA in hypoxic cells were gradually decreased. And then the inhibitory effects on MDR-1 and EPO mRNA, BSO concentrations reaching at 100 μM and 200 μM respectively, were shown statistical differences. Meanwhile, NAC could reduce the inhibition of BSO to MDR-1 and EPO mRNA. Furthermore, the expression of P-gp by MDR-1 translation, tested with western blotting, was also confirmed with the change of MDR-1 mRNA. Above experimental results were displayed in Figure 5 and Figure 6. It is therefore clear that redox micro-environment may influence the levels of target genes located at the downstream of HIF-1.

## Discussion

Among intracellular antioxidative factors, GSH is the tripeptide thiol L-γ-glutamyl-L-cysteinyl-glycine, a ubiquitous endogenous antioxidant. It plays an important role in maintaining intracellular redox equilibrium and in augmenting cellular defenses in oxidative stress [20,21]. In above antioxidant response, GSH is converted into glutathione oxidized disulfide (GSSG), which is recycled back to 2GSH by GSSG reductase, then forming what is known as a redox cycle. Under normal condition, the majority of glutathione is in the reduced form. Shifting redox equilibrium is in favor of a reducing or oxidizing state; that is in modification of the redox status in cells [22,23]. The γ-glutamylcysteine sythetase (γ-GCS) is the key rate-limiting enzyme synthesizing intracellular GSH, so intracellular GSH contents can be decreased by the inhibition of γ-GCS [24,25]. In the present study, our results showed that BSO, an inhibitor of γ-GCS, down-regulated the expression of GSH under hypoxia condition and the inhibitory effect was concentration-dependent. Conversely, intracellular GSH contents could be increased by adding NAC to medium. It is therefore apparent that the ratios of GSH and GSSG revealed the alterations of redox status in hypoxic cells by redox reagents pretreatment. Interestingly, we also noted that, as a precursor of GSH biosynthesis, NAC could not significantly decrease the suppression of GSH contents in the cells by 200 μm BSO pretreatment. One possibility was that, as high-concentration of BSO irreversibly suppresses the most parts of γ-GCS activities [24], the synthesis of GSH had been saturated without conspicuous increased by the addition of enzyme substrate.

Our following research showed that the down-regulation of HIF-1α in hypoxic cells by different concentrations BSO pretreatment, on the contrary, NAC could partly decrease the inhibitory effect. Similar to our results, the previous studies also showed that NAC, under chemical and physiological hypoxia, increased the expression of HIF-1α by changing cytoplasmic micro-environment redox state [26-28]. So it was clear that the redox status in hypoxic cells could influence the expression of HIF-1α protein. Combining the previous researches with our results, we considered the mechanism, the redox status influencing the expression of HIF-1α, as following: (i) The biosynthesis of GSH impose a reducing micro-environment, subsequently prolonging the half-life of HIF-1α and protracting its stability in cytosol and favouring its translocation [28]; (ii) GSH anti-oxidant system can effectively clear away free radicals and ROS that may suppress the expression of HIF-1α according to many previous studies [29,30]. However, it should be noted that some recent reports showed the opposite results, GSH contents being negative correlation with the levels of HIF-1α [31,32]. Based on other data, there could be the following factors contributing to these controversial phenomena: (i) Various cell types and experimental methods were used in different studies; (ii) The varies of GSH/GSSG equilibrium in different cells could exist in a certain range [23]. Excessive reducing status led to the extreme scavenging of the most of ROS and free radicals in hypoxic cells, but a bit of ROS generation from mitochondria possibly induced the expression of HIF-1α [33].

To further judge our finding, the expressions of MDR-1 and EPO, the down-stream target genes by HIF-1 promoting transcription in hypoxic cells, were observed in the present study. MDR-1 could encode P-gp at the membrane, effluxing chemtherapeutic reagents, to the resistance of tumor therapy. Under hypoxic condition, HIF-1 triggers the expressions of MDR-1 and EPO by binding to hypoxia-responsive elements (HRE) at positions -49 to -45 within the function regions of genes [34]. We found that the changing trend of MDR-1 and EPO was also coincident with the expression of HIF-1α. Consistent in our results, some previous studies using hypoxic DU-145 cells showed that intracellular redox status gave rise to the obvious alterations of MDR-1 expression [35,36]. Meanwhile, other study revealed that, under hypoxic condition, the concentration of EPO in plasma was enhanced by oral NAC treatment, the shifting of EPO could be further associated with an increased expression of HIF-1 [37]. Thus above findings also have another implication that regulating micro-environment redox status in hypoxic tumor cells may be beneficial to tumor chemotherapy by reduction of the expression of MDR-1 dependent upon HIF-1α.

Taken together, our results suggest that the alteration of intracellular micro-environment redox state can regulate the level of HIF-1α expression in hypoxic HepG2 cells. It is well known that the cellular and tissue's response to hypoxia is a central process in the pathophysiology of several diseases, including cancer, cardiovascular and respiratory disease, and so on [5,38,39]. The expression of HIF-1 plays an important role in above pathophysiological processes. It is valuable that the design of new type drugs is utilized to aim at the expression of HIF-1α through researching the mechanism of its expression in detail.

## Abbreviations

**HIF-1: **Hypoxia inducible factor; **BSO: **Buthionine sulphoximine; **GSH: **Reduce glutathione; **NAC: ***N-*acetylcysteine; **EPO: **erythropoietin.

## Competing interests

The authors declare that they have no competing interests.

## Authors' contributions

WSJ, YZJ: Conceived and designed the experiments;

ZLK, ZFS: Performed the experiments and analysed the data;

WKZ, GFC: Contributed reagents/material/analysis tools/.

All authors read an approved the final draft.
